# Supplementary figures and images for: Diagnostic accuracy of pelvic magnetic resonance imaging for the assessment of bone marrow involvement in diffuse large B-cell lymphoma
Source: PLoS One. 2021 May 27;16(5):e0252226. doi: 10.1371/journal.pone.0252226 (PMC8158887; doi:10.1371/journal.pone.0252226)

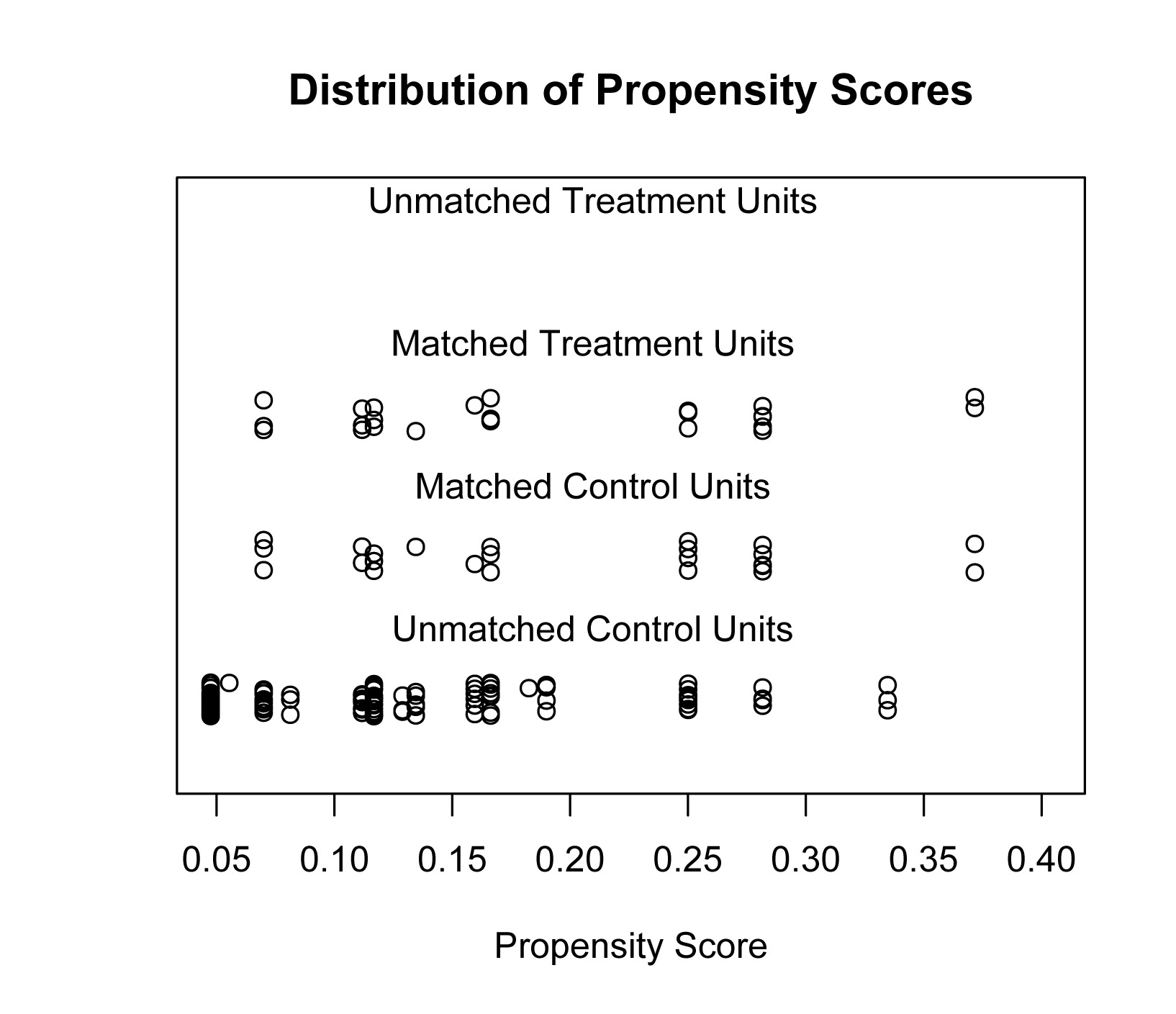

Supplement: S1 Fig — (TIF) [file pone.0252226.s001.tif]

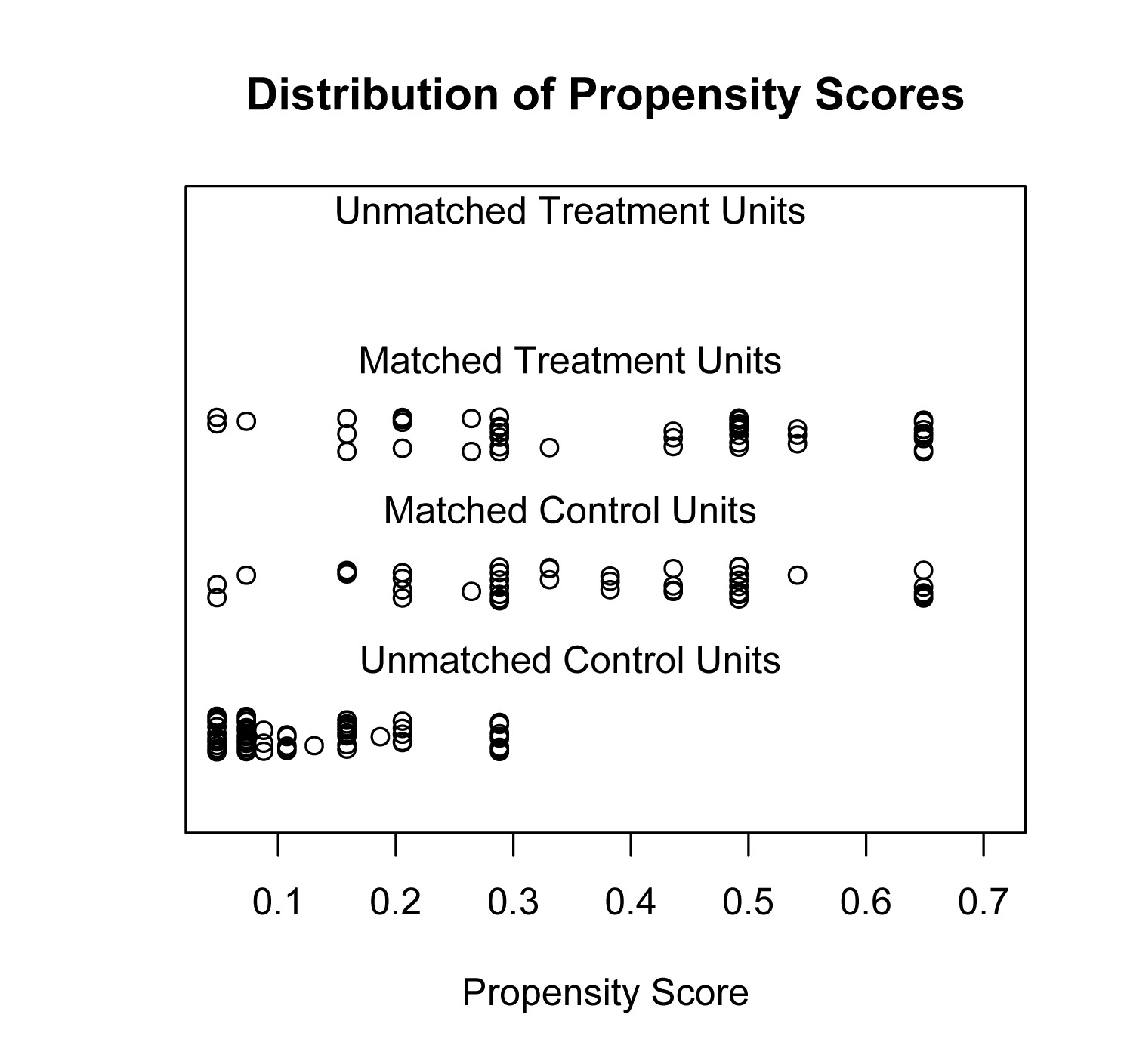

Supplement: S2 Fig — (TIF) [file pone.0252226.s002.tif]

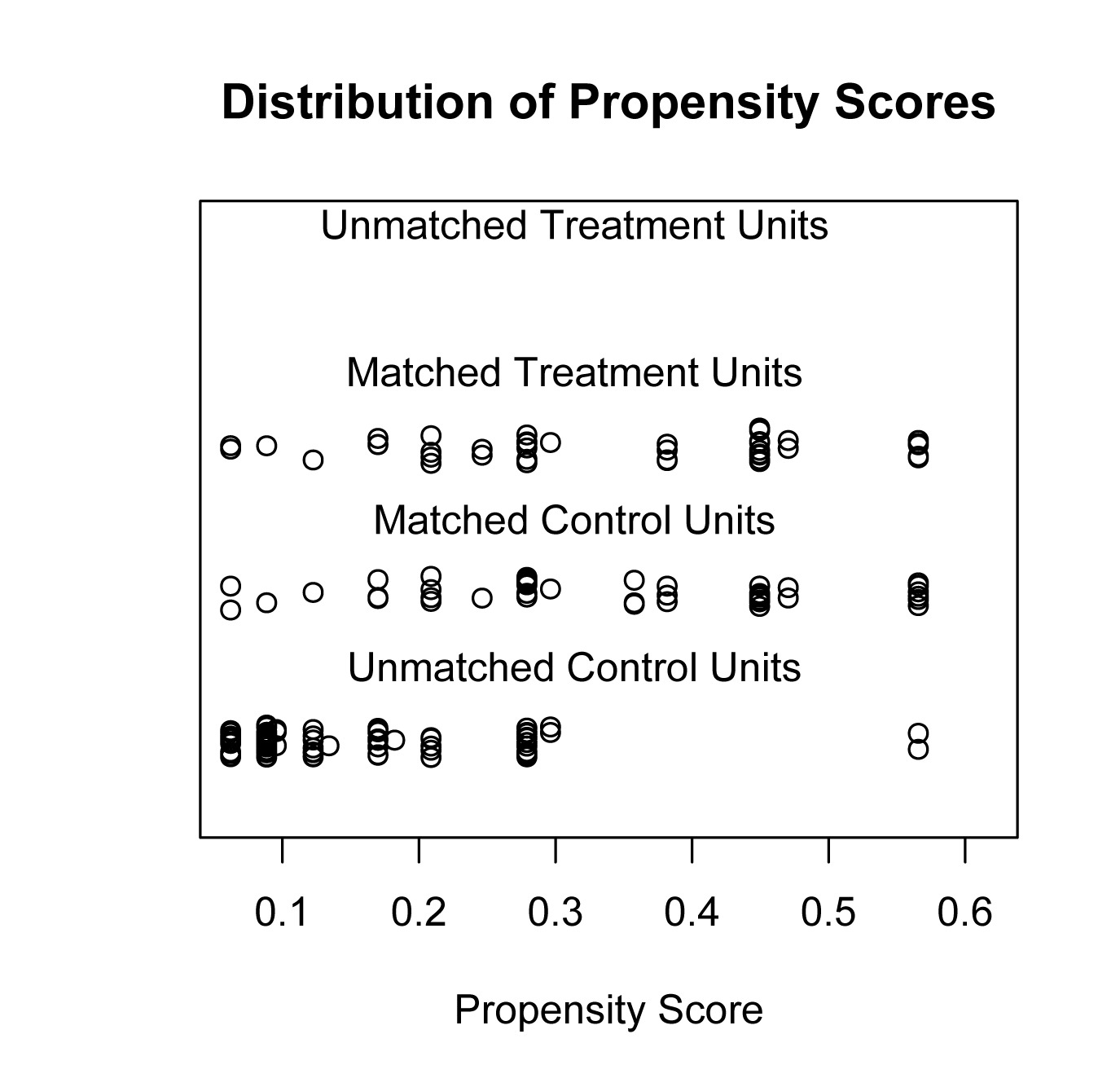

Supplement: S3 Fig — (TIF) [file pone.0252226.s003.tif]
